# Three new species of the leafhopper genus *Tambocerus* Zhang & Webb (Hemiptera, Cicadellidae) from southern China

**DOI:** 10.3897/zookeys.434.7712

**Published:** 2014-08-14

**Authors:** Ling Qu, Ren-Huai Dai

**Affiliations:** 1 Institute of Entomology, Guizhou University; The Provincial Key Laboratory for Agricultural Pest Management Mountainous Region, Guiyang, Guizhou 550025 P.R. China

**Keywords:** Auchenorrhyncha, taxonomy, morphology, China

## Abstract

Three new species, *Tambocerus dentatus*, *T. longicaudatus* and *T. robustispinus* spp. n. from southern China, are described and illustrated. A checklist and distribution to the *Tambocerus* species from China is provided together with a key for their separation.

## Introduction

The Oriental leafhopper genus *Tambocerus* was established by Zhang and Webb (1996) with *Selenocephalus disparatus* (Melichar, 1903) from Sri Lanka as its type species. It was placed in the tribe Selenocephalini (Zhang and Webb 1996) due to the transverse striations on the fore margin of the head but was assigned to the tribe Athysanini in the subfamily Deltocephalinae by Viraktamath (2012) according to the key to tribes of the subfamily Deltocephalinae (Zahniser and Dietrich 2008).

Zhang and Webb (1996) originally described the genus *Tambocerus* for two species, *Tambocerus disparatus* (Melichar) and *Tambocerus plumbeus* (Distant) from Sri Lanka. Rao (1996) added one new species from Indian, Shen et al. (2008) described four more species from China and Viraktamath (2012) described seven new species from the Indian subcontinent. So far, the genus *Tambocerus* contains 14 known species.

In this paper, we describe three new species, *Tambocerus dentatus* Qu & Dai, sp. n., *Tambocerus longicaudatus* Qu & Dai, sp. n. and *Tambocerus robustispinus* Qu & Dai, sp. n. from China, are described and illustrated. A checklist along with distribution records and a key to distinguish species of the genus from China are provided.

## Material and methods

Classification system follows that by Zahniser and Dietrich (2013), morphological terminology follows that by Zhang and Webb (1996), Shen et al. (2008) and Viraktamath (2012).

Type specimens of the new species are deposited in the Institute of Entomology, Guizhou University, Guiyang, China (GUGC) and one specimen of *Tambocerus robustispinus* sp. n. is deposited in the British Museum (Natural History), London, U.K. (BMNH).

## Taxonomy

### Genus Tambocerus

Taxon classificationAnimaliaHemipteraCicadellidae

Zhang & Webb

Tambocerus Zhang & Webb, 1996: 8–9; Shen et al. 2008: 242–249; Viraktamath 2012: 43–61.

#### Type species.

*Selenocephalus disparatus* (Melichar, 1903).

#### Remarks.

This genus can be differentiated from other genera by the following combination of characters: ocelli on margin close to eye, vertex slightly produced medially with anterior margin transversely striate, antennae located at level near to middle of eyes, connective Y-shaped with stem as long or longer than arms and aedeagal shaft laterally serrate.

#### Distribution.

Palearctic and Oriental region: Sri Lanka, Indian and China.

#### Checklist of the genus *Tambocerus* in China

*Tambocerus dentatus* Qu & Dai, sp. n.

Distribution. China (Guizhou Province).

*Tambocerus elongatus* Shen, 2008: 243–246, figs 1–7.

Distribution. China (Hubei, Hunan, Henan, Shaanxi, Guangxi, Hainan, Guangdong, Fujian, Sichuan, Anhui Provinces).

*Tambocerus furcellus* Shang & Zhang, 2008: 247–248, figs 15–21.

Distribution. China (Hunan Province).

*Tambocerus longicaudatus* Qu & Dai, sp. n.

Distribution. China (Guizhou Province).

*Tambocerus quadricornis* Shang & Zhang, 2008: 248, figs 22–28.

Distribution. China (Guangxi Province).

*Tambocerus robustispinus* Qu & Dai, sp. n.

Distribution. China (Guangxi and Yunnan Provinces).

*Tambocerus triangulatus* Shen, 2008: 246–247, figs 8–14.

Distribution. China (Shaanxi and Hainan Provinces).

#### Key to species of the genus *Tambocerus* from China (males)

**Table:** 

1	Pygofer caudal lobe rounded with dorsoposterior margin dentate (Fig. 10)	*Tambocerus dentatus* Qu & Dai, sp. n.
–	Pygofer caudal lobe produced (Figs 16, 23)	2
2	Pygofer lobe produced process-like (Figs 16, 23); apophysis of style not or slightly exceeding apex of connective (Shen et al. 2008: Fig. 27)	3
–	Pygofer lobe produced triangular shaped in lateral view, with caudal sclerotised cape-like region (Shen et al. 2008: Fig. 3); apophysis of style exceeding well beyond apex of connective (Shen et al. 2008: Fig. 6)	*Tambocerus elongatus* Shen
3	Posterior process of pygofer curved dorsally (Shen et al. 2008: Figs 17, 24)	4
–	Posterior process of pygofer either directed posteriorly or posteriorly and then ventrally (Figs 16, 23)	5
4	Aedeagal shaft without bifurcate apex, with pair of lateral subapical process (Shen et al. 2008: Fig. 28)	*Tambocerus quadricornis* Shang & Zhang
–	Aedeagal shaft with bifurcate apex, without processes (Shen et al. 2008: Fig. 23)	*Tambocerus furcellus* Shang & Zhang
5	Pygofer posterior process with acute apex (Fig. 23); aedeagal shaft with bifurcate apex (Figs 27–28)	*Tambocerus robustispinus* Qu & Dai, sp. n.
–	Pygofer posterior process with apex digitate and curved ventrally (Fig. 16); aedeagus with pair of lateral processes (Fig. 20)	6
6	Aedeagal shaft with processes distinctly longer than width of shaft (Figs 20–21)	*Tambocerus longicaudatus* Qu & Dai, sp. n.
–	Aedeagal shaft with processes similar in width to shaft (Shen et al. 2008: Fig. 14)	*Tambocerus triangulatus* Shen

**Figures 1–9.**
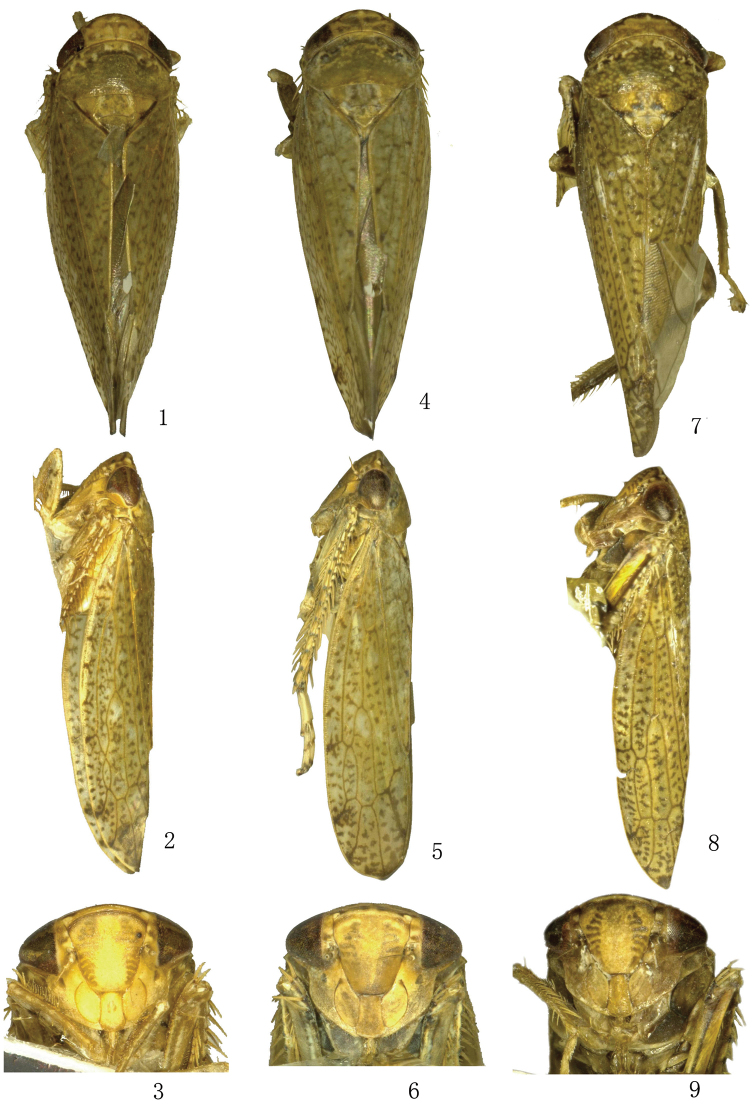
**1–3** *Tambocerus dentatus* Qu & Dai, sp. n. **4–6** *Tambocerus longicaudatus* Qu & Dai, sp. n. **7–9** *Tambocerus robustispinus* Qu & Dai, sp. n. **1, 4, 7** Dorsal view **2, 5, 8** Lateral view **3, 6, 9** Facial view.

**Figures 10–15.**
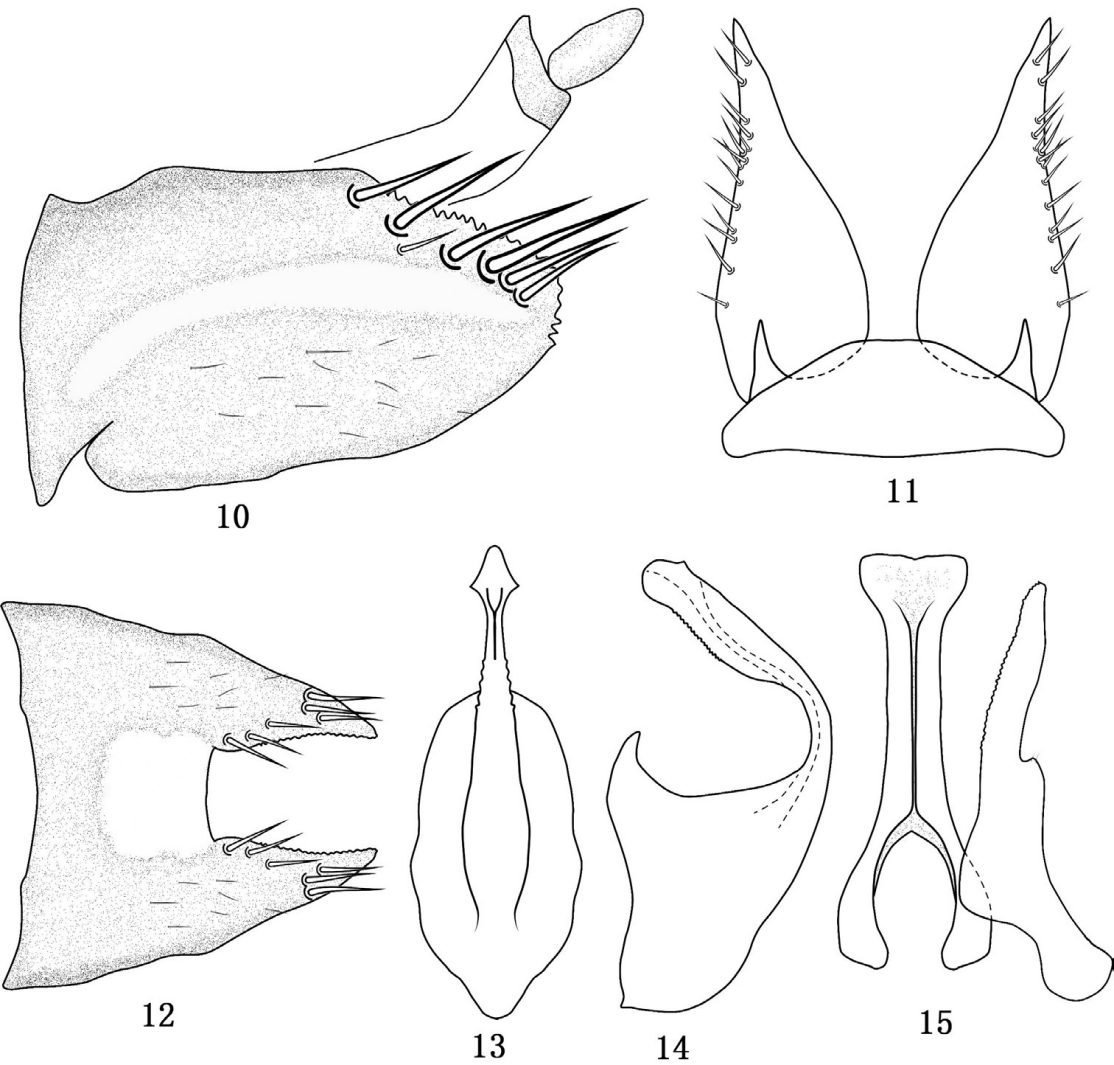
*Tambocerus dentatus* Qu & Dai, sp. n. **10** Pygofer in lateral view **11** Valve and subgenital fig in ventral view **12** Pygofer in dorsal view **13** Aedeagus in caudal view **14** Aedeagus in lateral view **15** Connective and style in dorsal view.

**Figures 16–22.**
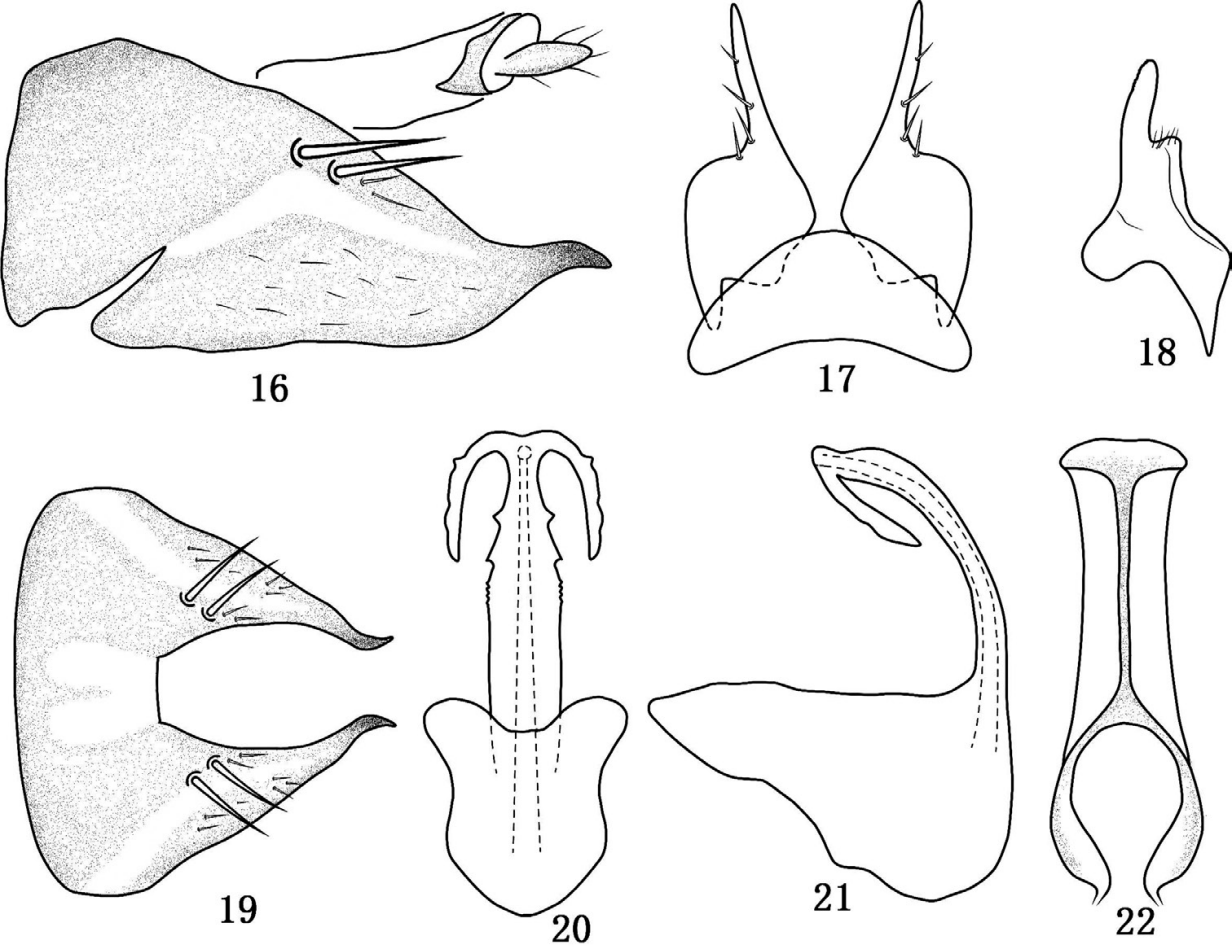
*Tambocerus longicaudatus* Qu & Dai, sp. n. **16** Pygofer in lateral view **17** Valve and subgenital fig in ventral view **18** Style in dorsal view **19** Pygofer in dorsal view **20** Aedeagus in front view **21** Aedeagus in lateral view **22** Connective in dorsal view.

**Figures 23–30.**
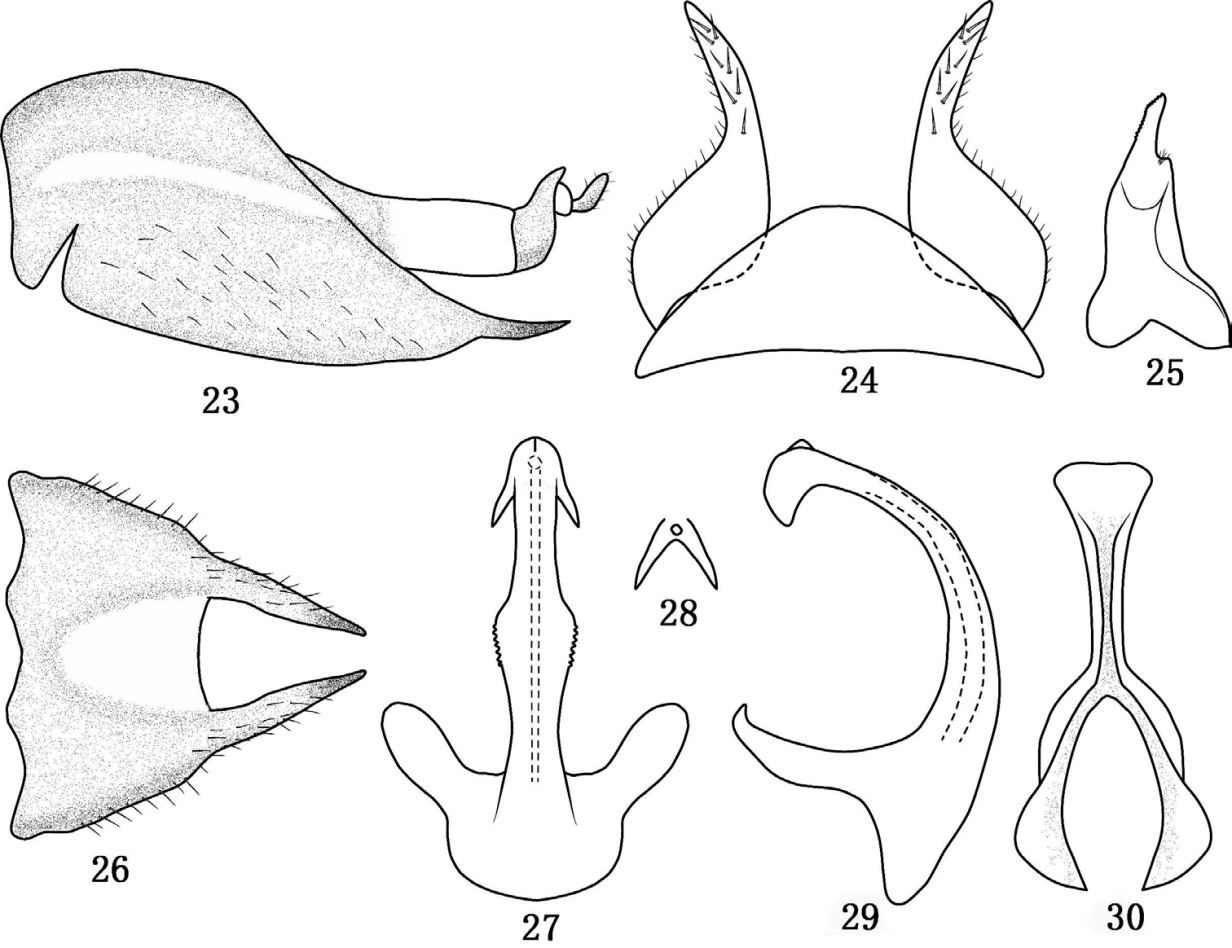
*Tambocerus robustispinus* Qu & Dai, sp. n. **23** Pygofer in lateral view **24** Valve and subgenital fig in ventral view **25** Style in dorsal view **26** Pygofer in dorsal view **27** Aedeagus in caudal view **28** Apex of aedeagus **29** Aedeagus in lateral view **30** Connective in dorsal view.

### Tambocerus dentatus

Taxon classificationAnimaliaHemipteraCicadellidae

Qu & Dai sp. n.

http://zoobank.org/814B98D5-DE3F-428A-AF16-36DAB803E210

Figs 1–3 , 10–15

#### Body length.

(including tegmina), ♂, 5.7 mm.

#### Description.

*Body colour*. Yellow-brown with dark brown markings and eyes fuscous. Vertex and pronotum with several light yellow blotches.

*Morphology*. Head (Fig. 1) including eyes nearly as wide as pronotum; vertex produced anteriorly with midlength 1.5 times length next to eyes. Fore tibia with dorsal setal formula 1+4.

*Male genitalia*. Pygofer (Figs 10, 12) with elongate lateral hyaline band and quadrate hyaline area on dorsal bridge; lobe with several long macrosetae dorsally and dorsal margin and apex serrated; valve (Fig. 11) semicircular; subgenital fig (Fig. 11) evenly tapered from base to acute apex with several short stout setae laterally; style (Fig. 15) relatively slender, with short and narrow lateral lobe, apophysis long, half length of style, serrate over inner margin; connective (Fig. 15) with stem twice length of arms; aedeagal shaft (Figs 13–14) abruptly constricted and curved dorsally near base in lateral view, distally laterally compressed with a medial subapical keel on the ventral surface, lateral margins serrate, with a short subapical processes on each side of ventral surface; phallobase well developed; dorsal apodeme short without lateral arms.

*Female*. Unknown.

#### Material examined.

Holotype, ♂, CHINA: Guizhou Province, Libo County, Wuyanqiao, 20. VII. 2011, collected by Zheng Weibin.

#### Distribution.

China (Guizhou Province).

#### Remarks.

This species externally resembles *Tambocerus elongatus* Shang and Zhang but can be separated from the latter by the male pygofer (Figs 10, 12) without process; the subgenital fig (Fig. 11) tapering from base to end; the aedeagal shaft (Figs 13–14) without depression at subapex in lateral view.

#### Etymology.

This species name is derived from the Latin word “*dentatus*”, referring to the dentate dorsal margin of the pygofer lobe.

### Tambocerus longicaudatus

Taxon classificationAnimaliaHemipteraCicadellidae

Qu & Dai sp. n.

http://zoobank.org/0BF009FF-5B6F-4BD1-B621-725C088A06D5

Figs 4–6 , 16–22

#### Body length.

(including tegmina), ♂, 6.3–6.5 mm.

#### Description.

*Body colour*. Body yellow-brown with dark brown patches and eyes fuscous or black.

*Morphology*. Head (Fig. 4) including eyes slightly narrower than pronotum; vertex with midlength 1.5 times length next to eyes. Fore tibia with dorsal setal formula 1+6.

*Male genitalia*. Pygofer (Figs 16, 19) with elongate lateral hyaline band and pair of lobe-like hyaline areas on dorsal bridge (Fig. 19), lobe with few long macrosetae dorsally at base, lobe well produced process-like with apex digitate and curved ventrally; valve (Fig. 17) triangular; subgenital fig (Fig. 17) abruptly narrowing at midlength, apical half slender with few short setae; style (Fig. 18) with short and broad subapical lobe, apophysis moderately long, digitate with inner margin dentate subapically; connective (Fig. 22) Y-shaped with stem one and a half times length of arms; aedeagal shaft (Figs 20–21) compressed dorsoventrally, dentate laterally over distal half to near apex, with pair of apical dorsolateral serrated processes approximately half length of shaft; phallobase well developed; dorsal apodeme with short robust arms.

*Female*. Unknown.

#### Material examined.

Holotype, ♂, CHINA: Guizhou Province, Suiyang County, Kuankuoshui National Natural Reserve, 5. VI. 2010, collected by Xing Jichun; Paratype, 1 ♂, CHINA: Guizhou Province, Suiyang County, Kuankuoshui National Natural Reserve, 8. VI. 2010, collected by Dai Renhuai and Li Hu.

#### Distribution.

China (Guizhou Province).

#### Remarks.

This species can be recognized by the extended and ventrally curved pygofer lobe (Figs 16, 19), sharply constricted subgenital figs (Fig. 17) at midlength and aedeagus (Figs 20–21) with pair of moderately long processes on apex.

#### Etymology.

The new species name is derived from the Latin words “*longus*” (long) and “*caudatus*” (tail), indicating the long pygofer extension.

### Tambocerus robustispinus

Taxon classificationAnimaliaHemipteraCicadellidae

Qu & Dai sp. n.

http://zoobank.org/4A27CF3B-F210-4BFC-A96A-FF2EC926A6E2

Figs 7–9 , 23–30

#### Body length.

(including tegmina), ♂, 6.1–7.0 mm.

#### Description.

*Body colour*. Yellow with dark brown spots and eyes fuscous or black. Pronotum with several light yellow irregular blotches in dorsal view.

*Morphology*. Head (Fig. 7) including eyes slightly wider than or nearly equal to pronotum; vertex slightly produced anteriorly with midlength 1.2 times length next to eyes. Fore tibia with dorsal setal formula 1+5 or 1+6.

*Male genitalia*. Male pygofer (Figs 23, 26) with elongate lateral hyaline band and semi crescent-shaped hyaline area on dorsal bridge, lobe (Figs 23, 26) with fine dorsal setae, lobe produced and tapered to acute apex with fine dorsal setae; valve (Fig. 24) triangular; subgenital fig (Fig. 24) gradually tapered to midlength thereafter with finger-like apex, with several short setae; style (Fig. 25) with short and narrow lateral lobe and moderately long apophysis dentate apically; connective (Fig. 30) with arms and stem similar in length; aedeagal shaft (Figs 27–29) cylindrical with serrated flange at midlength on each side, with bifurcate apically, short apical keel medially on ventral surface; phallobase narrow in lateral view; basal apodeme with moderately long widely spaced digitate arms.

*Female*. Unknown.

#### Material examined.

Holotype, ♂, CHINA: Guangxi Province, Wuming County, Damingshan National Natural Reserve, 15. V. 2012, collected by Li Hu; Paratypes, 6 ♂♂, same data as holotype; 7 ♂♂, CHINA: Guangxi Province, Wuming County, Damingshan National Natural Reserve, 19. V. 2012, collected by Fan Zhihua (one specimen deposited in BMNH); 2 ♂♂, CHINA: Guangxi Province, Longsheng County, Huaping National Natural Reserve, 19. V. 2012, collected by Yang Nannan and Fan Zhihua; 1 ♂, CHINA: Yunnan Province, Yuanyang County, Shangjiupai, 2. VIII. 2013, collected by Liu Yangyang.

#### Distribution.

China (Guangxi and Yunnan Provinces).

#### Remarks.

This species is similar to *Tambocerus furcellus* Shang and Zhang but can be distinguished by the male pygofer (Figs 23, 26) with acuminated and smooth process; the aedaeagus (Figs 27–29) with short spine ventrally.

#### Etymology.

This species is named for the stout apical processes of the aedeagal shaft.

## Supplementary Material

XML Treatment for Tambocerus

XML Treatment for Tambocerus dentatus

XML Treatment for Tambocerus longicaudatus

XML Treatment for Tambocerus robustispinus

## References

[B1] Rao KR (1996) Description of a new species *Tambocerus viraktamathi* (Homoptera: Cicadellidae) from India. Hexapoda 8(2): 85–88

[B2] Shen L Shang SQ Zhang YL (2008) Study of the leafhopper genus *Tambocerus* (Hemiptera: Cicadellidae) with four new species from China. Proceedings of the Entomological Society of Washington 110: 242–249. doi: 10.4289/0013-8797-110.1.242

[B3] Viraktamath CA (2012) Seven new species of the leafhopper genus *Tambocerus* (Hemiptera: Cicadellidae) from the Indian subcontinent. Zootaxa 3385: 43–61

[B4] Zahniser JN Dietrich CH (2008) Phylogeny of the leafhopper subfamily Deltocephalinae (Insecta: Auchenorrhyncha: Cicadellidae) and related subfamilies based on morphology. Systematics and Biodiversity 6: 1–24. doi: 10.1017/S1477200007002617

[B5] Zahniser JN Dietrich CH (2013) A review of the tribes of Deltocephalinae (Hemiptera: Auchenorrhyncha: Cicadellidae). European Journal of Taxonomy 45: 1–211

[B6] Zhang YL Webb MD (1996) A Revised Classification of the Asian and Pacific Selenocephaline Leafhoppers (Homoptera: Cicadellidae). Bulletin of the Natural History Museum (Entomology) 65(1): 1–103

